# Hepatitis C Virus-Specific Cell-Mediated Immune Responses in Children Born to Mothers Infected with Hepatitis C Virus

**DOI:** 10.1016/j.jpeds.2012.06.057

**Published:** 2013-01

**Authors:** Samer S. El-Kamary, Mohamed Hashem, Doaa A. Saleh, Sayed F. Abdelwahab, Maha Sobhy, Fatma M. Shebl, Michelle D. Shardell, G. Thomas Strickland, Mohamed Tarek Shata

**Affiliations:** 1 Department of Epidemiology and Public Health, University of Maryland School of Medicine, Baltimore, MD; 2 Department of Pediatrics, University of Maryland School of Medicine, Baltimore, MD; 3 Center for Vaccine Development, University of Maryland School of Medicine, Baltimore, MD; 4 Egyptian Company for Blood Transfusion Services (EgyBlood), VACSERA, Giza, Egypt; 5 Department of Public Health, Faculty of Medicine, Cairo University, Cairo, Egypt; 6 Department of Microbiology and Immunology, Faculty of Medicine, Minia University, Minia, Egypt; 7 Department of Public Health, National Liver Institute, Menoufia University, Shibin Elkom, Egypt; 8 Division of Digestive Diseases, Department of Internal Medicine, University of Cincinnati, Cincinnati, OH

**Keywords:** Anti-HCV, Antibodies to hepatitis C virus, CMI, Cell-mediated immunity, ELISPOT, Enzyme-linked immunospot assay, HCV, Hepatitis C virus, IFN-γ, Interferon-gamma, NS3/NS4, Nonstructural segments NS3, NS4a, and NS4b of the HCV genome, NS5, Nonstructural segment NS5 of the HCV genome, PBMC, Peripheral blood mononuclear cell, SFC, Spot-forming cell

## Abstract

**Objective:**

To investigate the association between hepatitis C virus (HCV)-specific cell-mediated immunity (CMI) responses and viral clearance in children born to mothers infected with HCV.

**Study design:**

A cross-sectional study of children from a mother-infant cohort in Egypt were enrolled to detect CMI responses to recombinant core and nonstructural HCV antigens (nonstructural segments NS3, NS4a/b, and NS5 of the HCV genome) using an interferon-gamma enzyme-linked immunospot assay. Children born to mothers with chronic HCV were enrolled into 3 groups: transiently viremic (n = 5), aviremic (n = 36), and positive control (n = 6), which consisted of 1 child with chronic HCV from this cohort and another 5 children with chronic HCV from a companion study. Children without HCV born to mothers without HCV (n = 27) served as a negative control group. Wilcoxon rank sum test was used to compare the magnitude of CMI responses between groups.

**Results:**

None of the 6 control children who were positive for HCV responded to any HCV antigen, and 4 (80%) of 5 children with transient viremia responded to at least one HCV antigen, compared with 5 (14%) of 36 and 3 (11%) of 27 children in the aviremic and negative control groups, respectively. Children with transient viremia elicited stronger responses than did negative controls (*P* = .005), positive controls (*P* = .011), or children without HCV viremia (*P* = .012), particularly to nonstructural antigens.

**Conclusions:**

HCV-specific CMI responses were significantly higher in magnitude and frequency among transiently infected children compared with those persistently infected. This suggests CMI responses may be associated with past viral clearance and can identify children at high risk of infection, who can be targeted for health education, screening, and follow-up.

Hepatitis C virus (HCV) is a blood-borne infection affecting nearly 130 million children and adults worldwide and is a major cause of liver cirrhosis and cancer.^1,2^ HCV is usually asymptomatic during initial infection and not diagnosed until complications develop, with increased mortality even among those without liver complications.^3,4^ Therapy is expensive and effective in only one-half of those who tolerate a prolonged therapeutic course.^5,6^ Controlling HCV infection will remain a challenge until an effective vaccine is developed, which, despite continuing efforts, remains elusive.^7^ Understanding the immune mechanisms of patients who successfully clear infection is essential to the design and development of an effective vaccine.

Spontaneous eradication of HCV occurs in 15%-50% of acute infections, and clearance is associated with specific immune responses.^4^ Although neutralizing antibodies have been identified, they are isolate-specific and poorly correlate with viral clearance.^8,9^ Cell-mediated immunity (CMI), however, has been shown to control HCV infection,^10^ and natural protective immunity has been demonstrated in both humans^11^ and chimpanzees.^12^

In the absence of antibodies to HCV (anti-HCV) or detectable viremia, HCV-specific CMI may represent the only biomarker of host contact with the virus and may provide a protective mechanism against chronic HCV infection.^11^ Understanding these immunologic responses—their longevity, magnitude, and breadth—is important for determining past exposure, understanding the natural history of HCV infection, and planning for prevention and treatment. If these responses are protective, then similar CMI responses need to be simulated by future vaccine candidates. Herein, we report CMI responses to recombinant HCV antigens representing both core and nonstructural proteins in a cohort of Egyptian children born to mothers infected with HCV from a highly endemic community and followed prospectively for 3-8 years after birth.

## Methods

This cross-sectional study evaluated HCV-specific CMI responses in a convenience sample of children enrolled from a larger prospective study of a mother-infant HCV transmission cohort in a high-prevalence area (24% infection) in Egypt^13-15^ conducted in 3 rural villages in the Nile Delta from 1997 until 2006. A total of 3457 mother-infant pairs (including 47 sets of twins), representing 3410 different women, consented to participate in the original study,^13-15^ and serum samples were collected prospectively from pregnant mothers and subsequently from both the mothers and their children at 2 months, 6 months, 1 year, and then annually thereafter. Viruses from a subsample of participants (28 samples) were subsequently sequenced^13^ and revealed that 27 (96%) were HCV genotype 4, which is quite close to the national prevalence of 90%,^16^ and reflects the highly homogeneous population of mothers in the villages from which they were enrolled.

Of the 1863 infants having complete baseline and follow-up data, 225 were born to mothers who were chronically infected (HCV RNA and anti-HCV positive), 7 were born to mothers who were only HCV RNA positive (total of 232 mothers with positive HCV RNA), and 97 were born to anti-HCV–positive and RNA-negative mothers; the remainder, 1534, were born to mothers who were both anti-HCV negative and HCV RNA negative.^13^ Of the children born to the 232 mothers with detectable HCV RNA, a total of 29 (12.5%) children had perinatal transmission, of which 14 were transient and cleared their viremia before their 1-year visit, and 15 had persistent viremia up to 2 years after birth, with 7 of these clearing their viremia by their third birthday. Thus, the total number of children with transient viremia was 21 (9%), and only 8 (3.5%) children had persistent HCV infection. All of the 14 infants with transient viremia lost their (presumably maternal) anti-HCV by 12-14 months, and the 7 who lost their viremia after 1 year lost their antibodies before their next annual visit.

Approximately 1 year after their last follow-up visit, we contacted a convenience sample of the first 50 mothers infected with HCV and 30 mothers not infected with HCV (based on the sample size calculation) from the 225 mothers who were chronically infected and 1534 mothers who were HCV RNA negative, and we preferentially approached mothers infected with HCV whose children had transient viremia. Of these 50 mothers infected with and 30 mothers not infected with HCV, 42 and 27 mothers consented to enroll their children into this cross-sectional study, respectively. The group of HCV-negative (seronegative, aviremic) children born to mothers not infected with HCV (seronegative, aviremic) served as a negative control group. The children not infected with HCV (seronegative, aviremic) who were born to mothers infected with HCV (seropositive, viremic) were divided into 2 groups: (1) seronegative, transiently viremic; and (2) seronegative, aviremic groups. One child infected with HCV born to a mother infected with HCV from this cohort and 5 children infected with HCV from a companion study^17^ served as a positive control group. The children ranged in age from 3 to 8 years. Given that this study was substantially different from the original mother-infant cohort study,^13-15^ we did not rely on the previous institutional review board approvals or consent form, and we sought and obtained separate approvals by the institutional review boards of the University of Maryland School of Medicine and the National Hepatology and Tropical Medicine Research Institute of the Egyptian Ministry of Health and Population. A new and separate consent form was signed by the parents who agreed to participate.

### Laboratory Tests for Anti-HCV Antibody and HCV RNA

All blood samples were tested for anti-HCV antibody with a third-generation enzyme immunoassay (Abbott Laboratories, Wiesbaden, Delknheim, Germany) and for HCV RNA using a qualitative direct nested reverse transcriptase–polymerase chain reaction as described previously.^13-15^ Laboratory personnel were given anonymized samples without knowledge of their source (whether infant maternal) or the visit number. Quantitative RNA testing was not performed, and transient infections were defined as detectable viremia by qualitative testing at least once after birth. Serum samples from 6 mother-infant pairs (5 with persistent infection and 1 with transient infection) were subsequently shipped to the University of Maryland for sequencing of the hypervariable region to (1) confirm viremia; (2) determine genotype; and (3) assess the correlation between the infant's and the mother's virus.^13^ All samples had detectable viremia and were predominantly genotype 4, and viruses from the 5 infants with infections that persisted beyond 1 or 2 years differed only by 0, 1, 2, 5, and 20 amino acids from the HCV isolated from the mother during her last trimester of pregnancy, with greater variation noted with increasing age of the child. The sample from the transiently infected child's virus differed by only 4 amino acids from his mother. This testing definitively confirmed the results of our laboratory in Egypt. The close correlation between the mother and infant's viral strains confirmed that the mother was the source of infection; the gradually increasing variation in infant sequences with increasing age reflects the expected viral mutation with time, confirming that the positive polymerase chain reaction samples were those of the infants and were closely correlated to their mothers.

### Recombinant HCV Antigens

We used superoxide dismutase–recombinant HCV protein antigens (courtesy of Chiron Corporation, Emeryville, California). These were derived from the HCV-1b sequence representing both structural segments, core (encoded; amino acids 2-120), and nonstructural segments of the genome, NS3/NS4 (NS3, NS4a, NS4b encoded; amino acids 1569-1931) and NS5 (NS5 encoded; amino acids 2054-2995). All HCV antigens were used at a final concentration of 10 μg/mL. At the time the study was conducted, recombinant HCV antigens derived from HCV genotype 4 were not available.

### Peripheral Blood Mononuclear Cell Isolation and Interferon-γ Enzyme-Linked Immunosorbent Assay

Peripheral blood mononuclear cells (PBMCs) were isolated from 5-8 mL of fresh blood samples using Ficoll-Hypaque density gradient centrifugation and were used fresh for the assay, with an average sample yield of 7.2 million cells per sample, or ∼1 million cells/mL. A modified mini-enzyme-linked immunosorbent spot (ELISPOT) assay was developed in our laboratories in Cairo and Cincinnati.^18,19^ The mini-ELISPOT plates (Whatman Unifilter, Florham Park, New Jersey) used less blood and were designed for CMI studies in children. The sensitivity of our mini-ELISPOT assay is comparable with the standard ELISPOT assay (unpublished data), and the assay procedures are described elsewhere.^17^ Briefly, freshly prepared PBMCs (1 × 10^5^ cell/well) were incubated in duplicate, and in a few cases, when enough cells were available, in triplicate, cultures in the mini-ELISPOT plates coated with anti–interferon (IFN)-γ antibody and incubated for 16 hours with or without recombinant HCV antigens at 10 μg/mL in complete RMPI-1640 medium. Positive controls were anti-CD3, staphylococcal enterotoxin B, and/or phytohemoglobin, and negative controls were *Escherichia coli* lysate, recombinant human superoxide dismutase, yeast lysate, and medium alone. The plate was incubated for 16-18 hours and then developed until spots appeared in the wells and then was rinsed with tap water. The number of spots per well was scored using an automated ELISPOT reader (Cellular Technology Ltd, Cleveland, Ohio). Functional viability was defined as having at least 1000 spot-forming cells (SFCs)/1 million PBMCs in response to anti-CD3 stimulation, staphylococcal enterotoxin B, and/or phytohemoglobin, with a mean of 4242 SFCs (SD 2345, range 1090-10 015). Averaged numbers of SFC in negative control wells (medium) were subtracted from antigen-stimulated wells to correct for spontaneous cytokine production, and the results were expressed per 1 million PBMCs. Positive HCV-specific IFN-γ response was defined as number of SFC greater than the mean response of the medium ±2 SDs, with a cut-off for a positive HCV antigen-specific response estimated to be >55 SFCs/10^6^.^17^

### Sample Size Calculation and Statistical Analyses

Sample size was calculated based on 2 groups of seronegative children: (1) children born to mothers infected with HCV; and (2) children born to mothers not infected with HCV. We expected that the former group would have a median HCV-specific CMI response rate of 60%, and the latter would have a median response rate of 20%. We calculated that 50 children of mothers infected with HCV and 30 children of mothers not infected with HCV would be sufficient to detect this difference with a type I error of 5% and a power of 80%, assuming a 20% dropout rate (ie, final sample sizes of 40 and 24, respectively). Statistical analysis was performed using Predictive Analytics Software Statistics 18.0.0 (formerly Statistical Package for the Social Sciences, Chicago, Illinois). The median and IQRs were used to describe the samples, and the Wilcoxon rank sum (Mann-Whitney U) test was used to compare the magnitude of CMI responses between the different child subgroups.

## Results

The age of the children ranged from 3 to 8 years (mean 4.9, SD 1.4 years), and 48% were females. There were 41 children not infected with HCV (seronegative, aviremic) born to mothers infected with HCV (seropositive, viremic), 1 child who was positive for HCV (seropositive, viremic) born to a mother infected with HCV, and 27 children not infected with HCV born to mothers not infected with HCV. Of the 41 children not infected with HCV, 5 had well-documented HCV transient viremia without antibody seroconversion (transiently viremic group), and 36 were aviremic children who never had a documented past episode of viremia. The 1 child infected with HCV born to a mother infected with HCV from this cohort and 5 children infected with HCV from a companion study^17^ served as a positive control group (total of 6 children). These 5 children infected with HCV were slightly older than the rest of the children enrolled into this study and ranged in age from 3.7 to 10.4 years (mean 6.7, SD 2.4 years), and 33% were female (Table I). The 27 children not infected with HCV born to mothers not infected with HCV were anti-HCV and HCV RNA negative at all time-points and served as a negative control group.

### Frequency and Breadth of HCV-Specific CMI Responses Using IFN-γ ELISPOT assay

The group with transient viremia had the greatest proportion of children who had a positive HCV-specific IFN-γ ELISPOT response to ≥1 of the 3 HCV antigens (4 [80%] responders of 5 children); the aviremic children born to mothers infected with HCV had 5 (13.9%) responders of 36 children; and the negative control group had 3 (11.1%) responders of 27 children (Table II). None of the 6 seropositive persistently viremic children elicited a positive response to any of the antigens tested. Table II shows the frequency of positive responses to ≥1, ≥2, or all 3 antigens stratified by group, with the fewest proportion of children being responsive to all 3 antigens simultaneously.

### Magnitude of HCV-Specific CMI Responses Using ELISPOT assay

The magnitude of HCV-specific CMI responses (total number of SFCs/10^6^ PBMCs) was highest overall among the transiently viremic in comparison with the aviremic (*P* = .012), the positive control (*P* = .011), and the negative control (*P* = .005) groups. The response to the core antigen among the transiently viremic (median, 30 SFC/10^6^ cells, IQR, 30-50 SFC/10^6^ cells) was significantly stronger than the aviremic (*P* = .018), positive control (*P* = .027), and negative control (*P* = .005) groups (Figure, A). The NS3/NS4 antigen (NS3, NS4a, NS4b) elicited the greatest magnitude of CMI responses among the transiently viremic (median, 840 SFC/10^6^ cells, IQR, 260-845 SFC/10^6^ cells) compared with the aviremic (*P* = .007), positive control (*P* = .035), and negative control (*P* = .001) groups (Figure, B), and the response to the NS5 antigen was stronger (median, 30 SFC/10^6^ cells, IQR, 15-360 SFC/10^6^ cells) compared with the positive control (*P* = .011) and the negative control (*P* = .015) groups but not the aviremic group (*P* = .117) (Figure, C).

The CMI responses displayed by the aviremic group were weaker than those of the transiently viremic group but were still stronger than those of the negative control group overall (*P* = .055), specifically in response to the NS3/NS4 antigen (*P* = .046) and the NS5 antigen (*P* = .055) but not to the core antigen (*P* = .347) (*P* values not shown in Figure).

## Discussion

This study demonstrates strong HCV-specific CMI responses among children born to mothers infected with HCV 3-8 years after birth. The children with transient viremia had the strongest IFN-γ responses to HCV antigens, particularly the NS3/NS4 antigens, with up to 80% responding to ≥1 HCV antigen. These strong, broad HCV-specific CMI responses are similar to those detected among adults whose infections resolved^20,21^ and whose loss has been associated with recurrent infection.^22^ Challenge studies in chimpanzees have demonstrated that similar vigorous HCV-specific immune responses can be experimentally induced by exposure to low doses of HCV.^23^ Similarly, some health care workers^24^ and injection drug users with HCV-specific CMI responses remain uninfected^25^ and develop broad T-cell responses that may protect them against subsequent HCV infection.^11^ This finding is not restricted to HCV but has been reported in HIV, where HIV-specific CMI responses without seroconversion or infection were detected among commercial sex workers at high risk for infection in Nairobi.^26^

The importance of CMI responses to resolving HCV infection in children is further strengthened by studies showing that humoral immunity was not necessary to clear infections in children^27,28^ and that maternal neutralizing antibodies transplacentally transferred to the fetus did not protect against vertical transmission of HCV.^29^ Reports of natural recovery from acute HCV infection by agammaglobulinemic twin children^27^ and a sustained virologic response after treatment of 2 brothers with X-linked agammaglobulinemia^28^ lend further evidence to the nonprotective role of anti-HCV compared with the crucial importance of CMI responses in resolving infections.

There is a paucity of studies investigating HCV-specific CMI responses in children.^17,30^ Our study assessed these responses several years after birth in children born to mothers infected with HCV. This is particularly important given that it represents the only immunologic marker of host contact with the virus, particularly after the maternally transmitted anti-HCV wanes. This is important in assessing past exposures, predicting the outcome of infection, and planning future care and follow-up. It is particularly relevant in Egypt, where universal hepatitis B infant immunization since 1992 and the low prevalence of HIV infection in the population (<0.1%)^31^ have made HCV the leading cause of blood-borne viral infection in children.^32^

Our study demonstrated that 80% of children with transient HCV infection and none of the children with persistent infection had HCV-specific CMI responses. These results compare with HCV-specific CMI responses in 14% of children born of children infected with HCV who had no detectable HCV RNA and 11% of children born of mothers who were not infected with HCV infections, most likely due to repeated low-dose exposures to other nonmaternal HCV-infected contacts in these high-prevalence villages.^17,19^ We believe these data represent a true immune reaction to HCV rather than cross-reactivity to other antigens (eg, influenza), which would have been similar in all groups.^33^ Interestingly, the presence of HCV-specific CMI responses in the absence of anti-HCV was also observed by Della Bella et al, where 71% of children born to mothers infected with HCV had circulating CD4^+^ lymphocytes reacting against HCV-specific antigens.^30^ In a study of adult blood donors, a similar proportion (83%) with a past resolved infection had a positive ELISPOT response.^34^ Similar HCV-specific CMI responses among seronegative individuals have been found in uninfected health care workers,^24,35^ spouses,^36^ household contacts,^19^ children living with siblings infected with HCV,^17^ prisoners,^37^ and injection drug users.^38^ These responses persisted for as long as 2 decades after the exposure.^39^

The strongest CMI responses were elicited in response to the NS3, NS4a, and NS4b antigen (amino acids 1569-1931), which corresponds to the nonstructural part of the HCV genome. This stretch of amino acids is important for viral replication^40^ and the processing of the HCV polyprotein to generate mature viral proteins, making it an ideal target for a protease inhibitor such as the HCV NS3/4A protease inhibitors telaprevir and boceprevir.^41,42^ It is possible that an early robust CMI response to this region inhibits HCV replication, allowing the host to successfully clear the virus, even before a humoral response is stimulated to produce anti-HCV.

A limitation of our study is that HCV-specific IFN-γ T-cell responses were determined only at 1 time-point, and hence we could not assess their subsequent protective properties. Also, in this study, we used HCV antigens derived from genotype 1 that were available to us at the time, in children with predominantly genotype 4 HCV exposure.^13^ However, genotype cross-reactivity tends to produce weaker CMI responses than antigens from the same genotype,^43^ thereby implying that if we had used genotype 4 antigens, the responses would likely have been stronger. Additionally, although the responses were most likely due to CD4^+^ T-cells given the nature of the antigens used, we could not verify these results by flow cytometry or using major histocompatibility complex class I–restricted peptide antigens to examine CD8^+^ responses due to the limited amount of blood available. However, our study was only a pilot study to assess whether HCV-specific CMI responses existed 3-8 years later in seronegative, aviremic children born to mothers infected with HCV. Although there were some CMI responders in the negative control and aviremic groups, this most likely reflects the background exposure to other infected household or community members as demonstrated in other studies by our group.^17,19^ Finally, we did not test other household members for HCV infection given that the parent study protocol was only designed to prospectively enroll pregnant women and their newborns. Hence, older siblings born prior to the study were not enrolled, nor were the fathers or other household members. In retrospect, enrolling these other household members would have been useful to determine whether other family members could have been sources of additional low-dose HCV exposures. However, it is still unlikely that the transiently viremic group acquired their infection from nonmaternal household or community contacts; otherwise, we would have expected the CMI responses to be similar in all groups. Furthermore, our group had previously shown that having a mother infected with HCV was the strongest predictor of HCV infection in the child.^44^ We recognize this limitation and hope that this pilot study will motivate additional studies that can focus on the subgroup of transiently infected children.

Using only antibody seroconversion as a marker for past infection in HCV studies has yielded the impression that children are at a low risk for past exposures and that less than half of those infected clear their infection. However, as seen in this study and others, IFN-γ ELISPOT CMI conversion has repeatedly proved to be a useful tool in detecting past exposure to the virus and can also be used to identify pediatric populations at high risk of infection, thereby providing them with health education, closer follow-up, and HCV screening. Furthermore, they could be preferentially targeted for preventive or prophylactic vaccination when HCV vaccines become available.

## Figures and Tables

**Figure:**
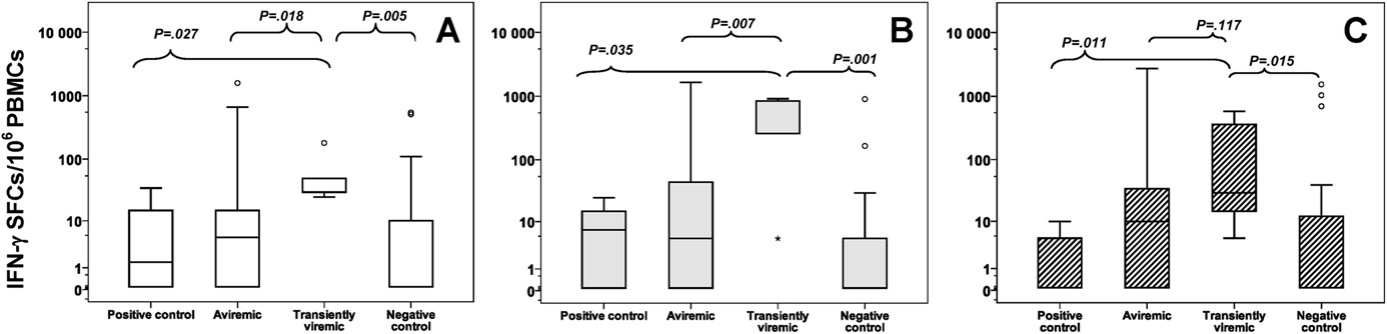
Median number of SFCs per 1 million PBMCs were quantified using an IFN-γ mini-ELISPOT assay and displayed by subgroup in response to stimulation with the 3 HCV-specific antigens. The *lower* and *upper borders* of each box encompass the IQR, the *horizontal line* inside the box represents the median, the *vertical lines* from the ends of each box extend to the extreme data points, and the *asterisks* represent outliers. The subgroups are: (1) positive control children with seropositive-persistent viremia (n = 6); (2) seronegative aviremic children born to mothers infected with HCV (n = 36); (3) children with seronegative transient viremia born to mothers infected with HCV (n = 5); and (4) a negative control group (children and their mothers not infected with HCV) (n = 27). In panels *P* values compare the magnitude of CMI responses of the transiently viremic group to the other subgroups for the **A,** core **B,** NS3/NS4 and **C,** NS5 antigens.

**Table I:** Age and sex of the children in the 4 groups

Group	Age, y			Sex (females)
Mean	SD	Range
Transient viremia (n = 5)	5.5	2.4	3.2-8.7	40.0%
Aviremic group (n = 36)	4.6	1.4	3.1-8.2	50.0%
Positive control group (n = 6)	6.7	2.4	3.7-10.4	33.3%
Negative control group (n = 27)	5.2	1.1	3.8-8.0	48.1%

**Table II:** Frequency of positive cell-mediated HCV-specific immune responses to ≥1, ≥2, or all 3 HCV antigens by group

Group	No. of HCV antigens eliciting a positive CMI response,∗ n (%)		
≥1 antigens	≥2 antigens	All 3 antigens
Transient viremia (n = 5)	4 (80.0)	2 (40.0)	0 (0.0)
Aviremic group (n = 36)	5 (13.9)	1 (2.8)	1 (2.8)
Positive control group (n = 6)	0 (0.0)	0 (0.0)	0 (0.0)
Negative control group (n = 27)	3 (11.1)	1 (3.7)	1 (3.7)

CMI responses were quantified using an IFN-γ mini-ELISPOT assay.

∗ Positive response was defined as number of SFCs greater than the mean response of the medium +2 SDs and >55 SFCs/10^6^ cells. The antigens used were core (amino acids 2-120), NS3/NS4 (amino acids 1569-1931), and NS5 (amino acids 2054-2995).
